# Operation for the Removal of a Large Artificial Toothplate from the Pharynx—Recovery

**Published:** 1856-06

**Authors:** 


### OPERATION FOR THE REMOVAL OF A LARGE ARTIFICIAL TOOTH-PLATE FROM THE PHARYNX---RECOVERY.

CASE UNDER THE CARE OF MR. COCK.

Mr. T. G., aged 22, a highly respectable tradesman at Dartford, was brought to Mr. Cock's residence on Thursday, January 17, by Mr. Martin, surgeon, of Dartford.

It appeared that for some time he had been wearing a false central incisor tooth fixed to a gold plate, which extended some distance on either side. The foreign body, which was subsequently removed from the pharynx, may be thus described: The plate formed the segment of a circle corresponding with the hard palate behind the incisor cuspidati and bicuspid teeth. The one extremity terminated in a slender clasp, with two points as sharp as needles, and encircling the bicuspid tooth; the other extremity formed a single sharp point. The anterior edge of the plate presented three acute angular projections, which corresponded with the inter-dental spaces; and from this margin also the false tooth formed a prominent projection. The extreme length of the plate—in other words, the *sector* of the circle—was an inch and five-eighths; while a line drawn from the edge of the tooth to the sector measured exactly one inch.

This plate had been swallowed by the patient during sleep, about 2 o'clock, A. M.; and Mr. Martin, finding that it had stuck in the gullet, and could neither be seen nor felt from the mouth, brought him up to Mr. Cock for further advice.

There could be no doubt that the foreign body had lodged in the cervical portion of the swallow, but its exact situation was not very clearly indicated. The pain and irritation, together with tenderness on pressure, all of which were very considerable, were referred to the top of the œsophagus, just below the larynx; but no projection indicating the precise locality of the plate could be detected from the exterior. He was able to swallow fluids, although in very small quantities and with great difficulty. His breathing was not impeded, but he had an irritating laryngeal cough.

Under these circumstances, Mr. Cock judged it most expedient to delay any active measures for extraction until the patient had recovered from the immediate effects of the accident and the fatigue and excitement of his journey. He was, therefore, advised to go into the Hospital, in order that every available means might be used; and he willingly agreed to this arrangement. In the course of the afternoon he was visited by Mr. Cock, who passed a bougie into the pharynx, and found a total obstruction about the lower edge of the larynx; in fact, just at the junction of the pharynx and œsophagus. A pair of strongly-curved forceps detected the plate, but it could neither be grasped nor moved from its position. As his respiration was unimpeded, and the pain quite bearable when kept at rest, it was determined to postpone further measures until the next day. A full dose of opium was given, as much fluid nourishment as he could get down was ordered, and he was furnished with ice to suck at his leisure.

On Friday, January 18, Mr. Cock saw him with Mr. Hilton. He was calm and tranquil, and had not suffered acutely except when pressure was made from the exterior, or when he attempted to swallow. It appeared very doubtful whether any fluid which he took into his mouth found its way into the œsophagus. Attempts were made with several instruments to grasp or dislodge the plate, but they all proved abortive, and it was found impossible to pass any instrument between the foreign body and the walls of the gullet, so as to get it below the obstruction. Mr. Cock at length, succeeded in introducing a flexible catheter, No. 5, which appears to have found its way between the horns of the clasp which formed one end of the plate. As a means of conveying fluid into the stomach had now been obtained, it was suggested that the action of vomiting might possibly alter the position of the plate, and render it more accessible from the mouth. A pint of milk was accordingly conveyed into his stomach, and then half-a-drachm of sulphate of zinc and a scruple of powdered ipecacuanha administered. Strange to say, not even a sense of nausea was produced, and the emetics were retained without producing the slightest constitutional effects. A mode of administering nourishment had, however, been obtained, and we could, therefore, afford to wait and take the chance of any favorable contingency. On Saturday, January 19, Mr. Cock made another attempt. Since the previous day he had twice fed the patient with milk, wine and, beef-tea; but the catheter was passed with great difficulty, and there was only one particular spot on the left side where it could be made to penetrate into the œsophagus. He was unable to swallow a drop of fluid by natural efforts, but derived great comfort from sucking ice. Mr. Cock attempted to pass a looped wire round the plate, and also manipulated with a flexible tube, from the extremity of which a pair of forceps could be projected, but no success could be obtained, and farther proceedings were laid aside for the present. On Sunday, January 20, no attempts were made, but the patient was fed three times through the catheter; the introduction of the instrument becoming more and more difficult each time. On Monday, January 21, Mr. Cock again met his colleagues. It was now imperative that some decisive steps to remove the foreign body should be taken, as the flexible catheter could no longer be passed, and the patient was beginning to feel seriously the effects of want of nourishment and rest. The position of the plate was pretty clearly ascertained. It was impacted either at the commencement of the œsophagus, or else just above, (where the œsophagus and the pharynx join). It was determined to cut down and open the gullet. Mr. Hilton assisted Mr. Cock in the operation.

The patient was placed on his back, with his head and shoulders slightly elevated. Chloroform was given, and he was soon quietly under its influence. An incision of about four inches in length was carried from the upper edge of the thyroid cartilage, nearly as far down as the sterno-clavicular joint; on the left side of which the platysma and cervical fascia were divided, bringing into view the carotid sheath and the omo-hyoideus muscle, which was thick and fleshy where it crossed the wound. This latter was divided, together with some filaments of the descendens lingualis nerve, and two or three small arteries, which were immediately tied to prevent as much as possible infiltration of blood into the cellular tissue. A little farther dissection laid bare distinctly the common carotid artery, the inner connexions of which were easily separated with the handle of the knife and the finger. It was considered to be an important object to separate completely the carotid artery from its internal attachments; and this having been accomplished, the vessel, together with the sterno-mastoid muscle, was drawn outwards and retained by retractors, and thus rescued from injury or molestation, while the further steps of the operation were carried on, the object of which was to reach the upper portion of the œsophagus.

The thyroid body was now exposed by dividing a few of the external fibres of the sterno-hyoid and sterno-thyroid muscles, and the dissection was continued along the outer surface of the gland backwards towards the spine. The tissues were separated partly by the handle of the knife, partly by the blade. An artery, probably a branch of the superior thyroideal, was divided where it crossed the upper part of the wound, bled freely, and was secured with some difficulty. A larger vessel, probably the inferior thyroideal, was seen running across lower down, but escaped without injury. The larynx and trachea were gently drawn over towards the right side, so as to widen the large wound which gaped along the side of the neck.

The œsophagus was reached by following round the surface of the thyroid body, which completely covered and concealed the trachea.

About two inches of the gullet could now be traced with the finger, but no projection indicating the presence of the foreign body could be felt. It therefore seemed tolerably certain that the plate had not descended into the œsophagus, and must be lodged in the lower part of the pharynx. With some difficulty, by tilting the larynx a little forwards and over to the left, the finger was passed behind it, that is, between the pharynx and the vertebræ, and the body was now obscurely felt exactly behind the cricoid cartilage, protected as it were by the inferior course of the thyroid. The point of the knife was now brought to bear on what appeared to be the most prominent part, which proved to be the single tooth, and the grating sensation of the blade indicated that the pharynx was opened, and the foreign body reached.

The white tooth, in fact, became visible at the bottom of the wound; and, being grasped with a pair of forceps, the opening into the pharynx was dilated upwards and downwards with a blunt pointed bistoury. After a little manipulation, one end of the plate was disentangled from its attachments and brought out of the wound, but the entire body was not extricated until a further slight division of the walls of the pharynx had been made. This, however, was soon accomplished, with the assistance of Mr. Hilton, who cut along the edge of the gold plate, while Mr. Cock gently withdrew it with one hand, and protected the parts with the fingers of the other. The patient was carried to his bed, and cold water applied to the wound, no means being used to bring the edges together. On recovering from the effects of the chloroform, he seemed to have suffered but little from the operation, expressed himself as comfortable and free from pain, and returned eagerly to his former occupation of sucking ice. An enema of beef-tea and wine was thrown up, as he had no nourishment since the previous day. In the evening he complained of great exhaustion, or rather sense of starvation, and Mr. Cock gave him nourishment through the catheter, and a full dose of opium.

January 22.—Was free from all untoward symptoms, and only complaining of an empty stomach. He was fed with milk and beef-tea three times. Sucking ice was a great luxury, although he believed that none of it passed into the œsophagus, and, as far as could be ascertained, no water found its way out by the wound. On the third day, January 24, Mr. Cock introduced the common œsophagus feeding-tube, which passed readily, without pain or obstruction. He has since been regularly fed by his dresser, Mr. Dyer, at first, three times, but afterwards, at his own request, four times in twenty-four hours. He is always ready, indeed eager, for his meals, and receives them with great enjoyment. His diet consisted of beef-tea, brandy, and egg, arrow-root, with milk or wine. Notwithstanding this nourishment, of which he swallowed about four pints in the twenty-four hours, he was evidently losing flesh and strength. Accordingly, Mr. Cock ordered as much pounded meat to be mixed with the beef-tea as could be made to pass through the tube, and directed an ounce to an ounce and a half of cod-liver oil to be given at each meal. He takes an opiate every night, but the quantity is undergoing gradual diminution.

February 5.—The increase of nutriment, or the oil, or both, have produced a decided improvement in his appearance, and he expresses himself as feeling stronger and better. His spirits have all along been good and hopeful.

The wound has looked healthy from the first, and has now contracted to half its original size. Since the operation, nothing has been swallowed by natural deglutition, and he is very unwilling to make the attempt, as it causes considerable pain, and a sensation as if the wound was being rent open. He does not appear to swallow his saliva.

Had the foreign body been lodged in the upper part of the œsophagus, its extraction would probably have been more easily accomplished; but the protection which was afforded by the cricoid cartilage in front, and the posterior edge and inferior course of the thyroid, which, as it were, overlapped it at the side, rendered the access to it difficult and tedious, and materially complicated the operation.—*London Medical Times.*

